# Cortical circuits linking peripheral inflammation to affective states

**DOI:** 10.3389/fncel.2026.1951226

**Published:** 2026-10-08

**Authors:** Dahae J. Jun, Shi Tang, Emilia Favuzzi

**Affiliations:** 1 Department of Neuroscience, Kavli Institute for Neuroscience, Yale University School of Medicine, New Haven, CT, United States; 2 Wu Tsai Institute, Yale University, New Haven, CT, United States

**Keywords:** anxiety, cytokines, insular cortex, interoception, neuroimmune communication, prefrontal cortex, vagus nerve

## Abstract

Affective disorders, such as anxiety and depression, are more prevalent in individuals with inflammatory diseases than in the general population. This comorbidity suggests biological links between immune activation and affective dysregulation. Indeed, there is extensive literature on the impact of inflammation on brain function. Peripheral immune challenges activate subcortical and brainstem regions that regulate emotions and physiological homeostasis. However, cortical regions are also engaged during peripheral inflammation, and cortical dysfunction is a central feature of affective pathology. Drawing primarily on mechanistic work in rodents, we review how peripheral inflammation reaches the cortical circuits that govern emotion and how it is translated into affective changes. This communication begins with two complementary routes by which immune signals reach the brain: neural and humoral pathways. In the neural pathway, cytokine-selective vagal afferents preserve the anatomical origin of inflammation and relay it to the brainstem and downstream regions. In the humoral pathway, circulating mediators act on circumventricular organs and across the blood-brain barrier. Cortical regions, such as the prefrontal cortex, insular cortex, and primary somatosensory cortex, transform these signals into altered behavior. This occurs through shifts in excitatory/inhibitory balance, top-down control of the amygdala, and interoceptive predictions of bodily state. We integrate these studies into a circuit-level model in which inflammation biases cortical processing toward threat, driving the transition from adaptive responses to maladaptive affective dysregulation. Defining which immune signals engage which circuits, and with what specificity, remains a central open question and a promising route toward targeting the affective consequences of inflammatory disease.

## 1 Introduction

When the body initiates an immune response to infection or injury, the inflammatory signals are not confined to the body but also influence the brain. Large-scale epidemiological data show that patients with chronic inflammatory or allergic diseases experience anxiety or depression at roughly 1.5 to 2 times the general population (Lu et al., 2018; Liu et al., 2023; Thiele et al., 2026). Notably, anxiety often precedes the diagnosis of inflammatory disease (Mikocka-Walus et al., 2016; Marrie et al., 2017, 2019; Bisgaard et al., 2023; Howren et al., 2024). This pattern points to a fundamental, yet incompletely understood, link between peripheral immune activity and the neural circuits that govern affective state. This connection is not incidental, but evolutionary. The immune and emotional systems are tightly coupled survival mechanisms: emotion assigns salience to threats and coordinates the physiological and behavioral responses required for effective immune defense.

Here, we use affective state as the broad term for the emotional consequences of peripheral immune activation. We further distinguish adaptive and maladaptive affective responses. Adaptive affective responses, such as hypervigilance, anticipatory anxiety, and avoidance of re-exposure, support defense and recovery. When this system becomes dysregulated, the same immune-to-brain signaling that once promoted survival drives the maladaptive persistence of altered affective states beyond the inflammatory episode. Although we include correlational human data, most evidence reviewed here comes from rodent models, which permit causal inference.

## 2 Body-to-brain routes: how peripheral inflammation is communicated to the brain

A central question linking inflammation and affective states is how signals originating in the periphery gain access to the brain and influence the networks that govern affect. Peripheral inflammatory signals reach the brain through two main routes: the neural and humoral pathways (Figure 1). Immune mediators initiate both, what distinguishes them is whether sensory afferents relay the signal or it reaches the brain directly.

**FIGURE 1:**
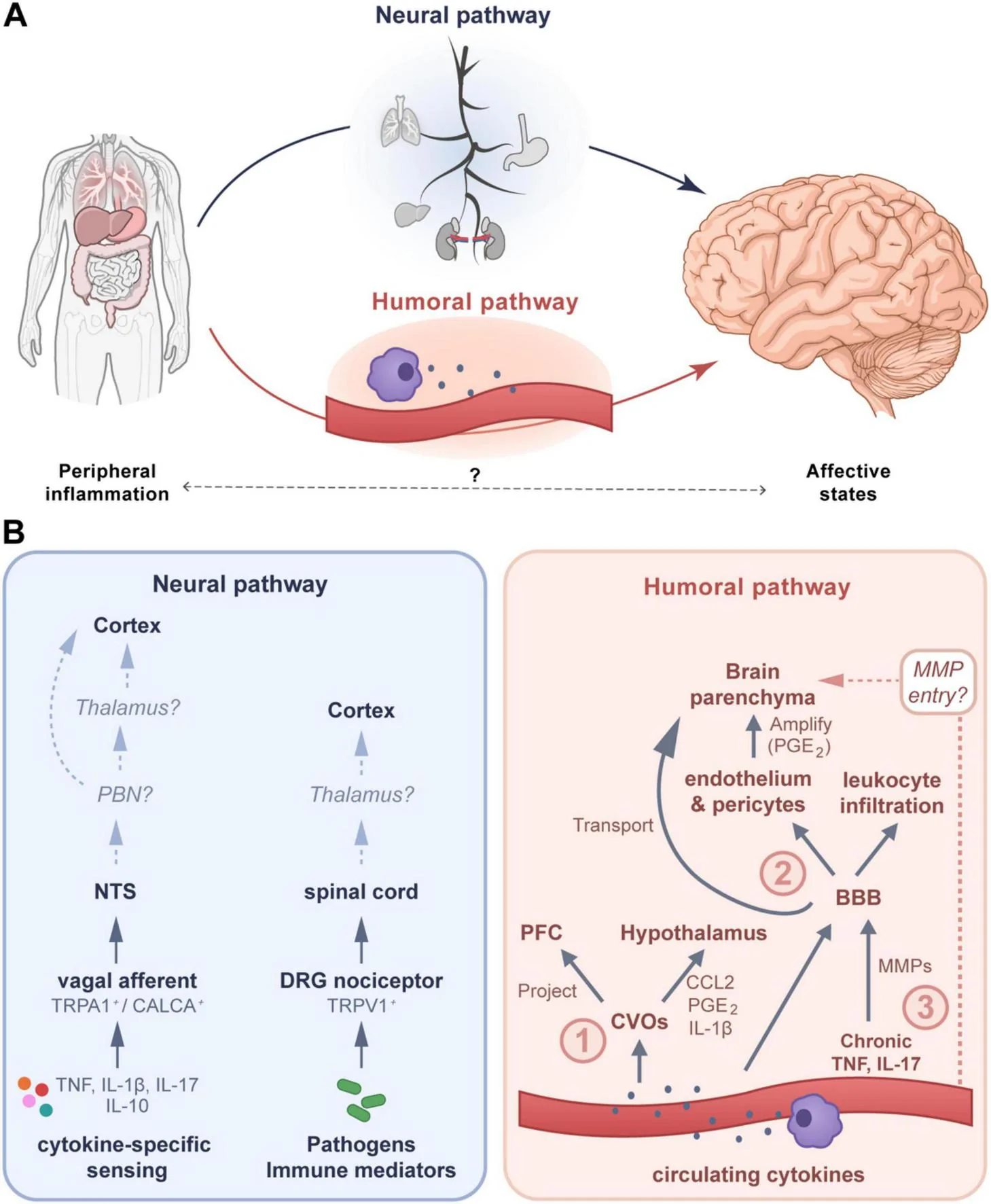
Peripheral inflammation reaches the brain through neural and humoral routes to influence affective behavior. **(A)** Overview of body-to-brain signaling. Inflammation arising in peripheral organs during infection or injury is communicated to the brain along two complementary routes. In the neural pathway (top), sensory neurons innervating peripheral organs, most notably vagal afferents, detect inflammatory mediators and relay this information rapidly to the brain. In the humoral pathway (bottom), circulating immune cells release cytokines (dots) that travel in the bloodstream to act on the brain directly. Both routes engage central circuits that regulate emotion and are implicated in affective dysregulation. The dashed arrow (⇠⇢) indicates that, although peripheral inflammation is epidemiologically associated with these states, a direct causal link remains unproven. **(B)** Mechanisms of the two routes. Full black arrows denote experimentally supported steps; dashed arrows and gray italicized labels followed by a question mark denote proposed or not-yet-resolved connections. In the *neural pathway* (blue), cytokines released by peripheral immune cells – pro-inflammatory tumor necrosis factor (TNF), interleukin-1β (IL-1β), and interleukin-17 (IL-17), and anti-inflammatory interleukin-10 (IL-10) – are detected by distinct vagal afferent populations expressing transient receptor potential ankyrin 1 (TRPA1) or calcitonin-related polypeptide alpha (CALCA), which project to the nucleus of the solitary tract (NTS); the NTS likely relays the signal via the parabrachial nucleus (PBN) and thalamus to the cortex. In parallel, dorsal root ganglion (DRG) nociceptors expressing transient receptor potential vanilloid 1 (TRPV1) directly detect pathogens and immune mediators and signal through the spinal cord and thalamus to the cortex. In the humoral pathway (red), circulating cytokines reach the brain by three routes: (1) at circumventricular organs (CVOs), which lack a complete blood-brain barrier (BBB), non-neuronal cells sense circulating cytokines and secrete C-C motif chemokine ligand 2 (CCL2), prostaglandin E_2_ (PGE_2_), and IL-1β to activate hypothalamic neurons (Osterhout et al., 2022), while CVOs also project directly to the prefrontal cortex (PFC); (2) across the intact BBB, endothelial cells and pericytes act as active transducers that amplify and transport the inflammatory signal into the brain parenchyma via secondary messengers such as PGE2; and (3) under strong or chronic inflammation, TNF and IL-17 induce matrix metalloproteinases (MMPs) that increase BBB permeability and permit leukocyte infiltration, while circulating MMPs may themselves enter the brain parenchyma to act directly on neural tissue.

### 2.1 The neural pathway

Immune information is conveyed from the periphery to the brain by two major classes of sensory neurons: spinal afferents with cell bodies in the dorsal root ganglia (DRG) and cranial afferents with cell bodies in the nodose ganglia of the vagus nerve. These neurons provide a rapid, topographically organized mechanism for monitoring physiological threats and coordinating responses to infections or injury.

Immune molecules and pathogens can directly activate DRG sensory neurons, particularly nociceptors that express transient receptor potential vanilloid 1 (TRPV1). TRPV1 nociceptors specialize in transmitting pain, a cardinal feature of inflammation, but can also directly sense infectious agents, including bacterial components and toxins (Julius, 2013; Chiu et al., 2013). The central axons of DRG neurons terminate in the spinal cord and relay “immune information” to the brain presumably via canonical spinothalamic pathways (Dum et al., 2009; Koch et al., 2018; Choi et al., 2020).

Decades of work have established that vagal sensory neurons also respond to immune mediators. The first evidence came from electrophysiological recordings and c-Fos staining after peripheral administration of interleukin (IL) 1β (Niijima, 1996; Goehler et al., 1998; Ek et al., 1998). Vagal neurons, however, do not respond indiscriminately: genetically distinct populations of vagal neurons detect specific mechanical and chemical stimuli arising from individual organs, such as stretch or nutrients (Chang et al., 2015; Williams et al., 2016). Cytokine sensing appears to follow the same logic. Steinberg and colleagues found that tumor necrosis factor (TNF) and IL-1β evoke distinct afferent neurograms (Steinberg et al., 2016). More recently, *in vivo* calcium imaging studies refined this picture. Jin and colleagues showed that pro- and anti-inflammatory cytokines activate two discrete, non-overlapping populations of nodose neurons, expressing Calcitonin Related Polypeptide Alpha (CALCA) and transient receptor potential ankyrin 1 (TRPA1), respectively (Jin et al., 2024). Huerta and colleagues further found that individual neurons produce stereotyped, concentration-dependent responses that are cytokine-specific in amplitude and kinetics, with some tuned to a single cytokine (IL-1β, TNF, or IL-10) and others encoding several cytokines through distinct activity patterns (Huerta et al., 2025). Together, these studies establish the vagus nerve as a critical sensory conduit for peripheral immune information. How the vagus detects cytokines, however, remains incompletely resolved. Cultured nodose neurons show surface expression of TNF and IL-1β receptors (Steinberg et al., 2016), yet IL-1 receptor binding sites have also been localized to the glomus cells of vagal paraganglia (Goehler et al., 1997). Whether vagal neurons themselves express cytokine receptors *in vivo* or instead respond to signals from neighboring stromal cells, and how they achieve cytokine specificity, remain unresolved.

Vagal afferents project primarily to the nucleus of the solitary tract (NTS), the first central site where organ-specific immune signals converge. TRPA1^+^ and CALCA^+^ afferents synapse monosynaptically onto dopamine β-hydroxylase-expressing (DBH^+^) neurons in the caudal NTS, which bidirectionally regulate peripheral immune responses and function as a central rheostat that balances pro- and anti-inflammatory reflexes (Jin et al., 2024). Repeated allergen exposure activates these same neurons in a vagus nerve-dependent manner, and they relay this signal through the nucleus ambiguus to drive airway hyperreactivity (Su et al., 2024). Thus, the NTS not only receives immune information but returns motor commands to the affected organ. Through projections to the hypothalamus, the NTS also transforms immune signals into sickness behavior. Lipopolysaccharide (LPS), a bacterial mimic, activates ADCYAP1^+^ neurons, overlapping with the DBH^+^ population, that are both necessary and sufficient for LPS-induced anorexia, adipsia, and lethargy (Ilanges et al., 2022). Since individual NTS neurons are tuned to detect mechanosensory and chemosensory signals viscerotopically (Ran et al., 2022), whether inflammatory signals are sensed according to similar principles is an important avenue for future work.

### 2.2 The humoral pathway

Not all immune signals travel the neural route. Soluble circulating mediators can reach the brain via circumventricular organs (CVOs) or direct transport through the blood-brain barrier (BBB). The BBB is a specialized endothelial structure that protects the brain by tightly regulating its ionic composition and excluding circulating toxins (Abbott et al., 2010). CVOs lack a complete BBB and are permeable to circulating inflammatory mediators even under homeostatic conditions (Johnson and Gross, 1993; Salvador et al., 2021). Their fenestrated vasculature and efferent projections make CVOs well suited to transduce systemic immune signals into neural activity. Osterhout and colleagues showed that two hallmarks of sickness behavior, fever and appetite suppression, are driven by neurons in the ventral medial preoptic area (VMPO) of the hypothalamus, which are engaged by a local paracrine mechanism: non-neuronal cells at the adjacent organum vasculosum of the lamina terminalis, a preoptic CVO, detect circulating immune signals and activate nearby VMPO neurons (Osterhout et al., 2022). In addition to this local paracrine action, CVOs project directly to regions involved in affective regulation, including the cortex. As such, they form a key anatomical link between circulating cytokines and behaviors associated with affective dysregulation (Mancz et al., 2026).

Beyond CVOs, even the intact BBB actively transduces peripheral immune signals and governs cytokine access to the broader brain parenchyma: endothelial cells and pericytes respond to circulating cytokines by producing secondary messengers such as prostaglandins, which diffuse into the parenchyma and propagate the inflammatory signal into neural tissue (Ek et al., 2001; Vasilache et al., 2015). Strong or prolonged inflammation shifts this balance from signal transduction toward structural compromise. Pro-inflammatory cytokines, including TNF and IL-17, induce matrix metalloproteinase activity (MMP-2 and MMP-9), driving tight junction remodeling, increased permeability, and ultimately leukocyte infiltration into the brain (Agrawal et al., 2006; Varatharaj and Galea, 2017). These effects are amplified through sphingosine-1-phosphate receptor signaling and chemokine-MMP interactions (Song et al., 2015; Yanagida et al., 2017). Consistently, inflammatory states such as sepsis are associated with increased BBB permeability and elevated central cytokine levels (Comim et al., 2011; Barichello et al., 2023). Under chronic stress, MMP8, a myeloid-derived proteinase elevated in major depressive disorder, can enter the nucleus accumbens, remodels the extracellular space, and alters behavior (Cathomas et al., 2024). Whether MMP entry also occurs during immune activation remains to be determined.

## 3 Cortical circuits linking peripheral inflammation and affective states

Research on how the brain responds to peripheral inflammation has focused primarily on sickness behavior and the subcortical circuits that drive it (Bin et al., 2023; Sullivan and Dulac, 2025). The cortical circuits that generate affective states are engaged as well, but how immune information ascends from the body to the cortex is only partly resolved. In this section, we review how inflammation alters cortical circuits that govern affective states, noting for each region which route is established and which is presumed (Figure 2). Most of this literature concerns interleukins. Other immune mediators and cell types shape cortical function in non-affective contexts, such as monocyte-derived TNF-α in learning (Garré et al., 2017), but their contribution to affective circuits is untested. Neuroendocrine and metabolic routes engaged by inflammation are beyond the scope of this review and have been reviewed elsewhere (Dantzer et al., 2008).

**FIGURE 2:**
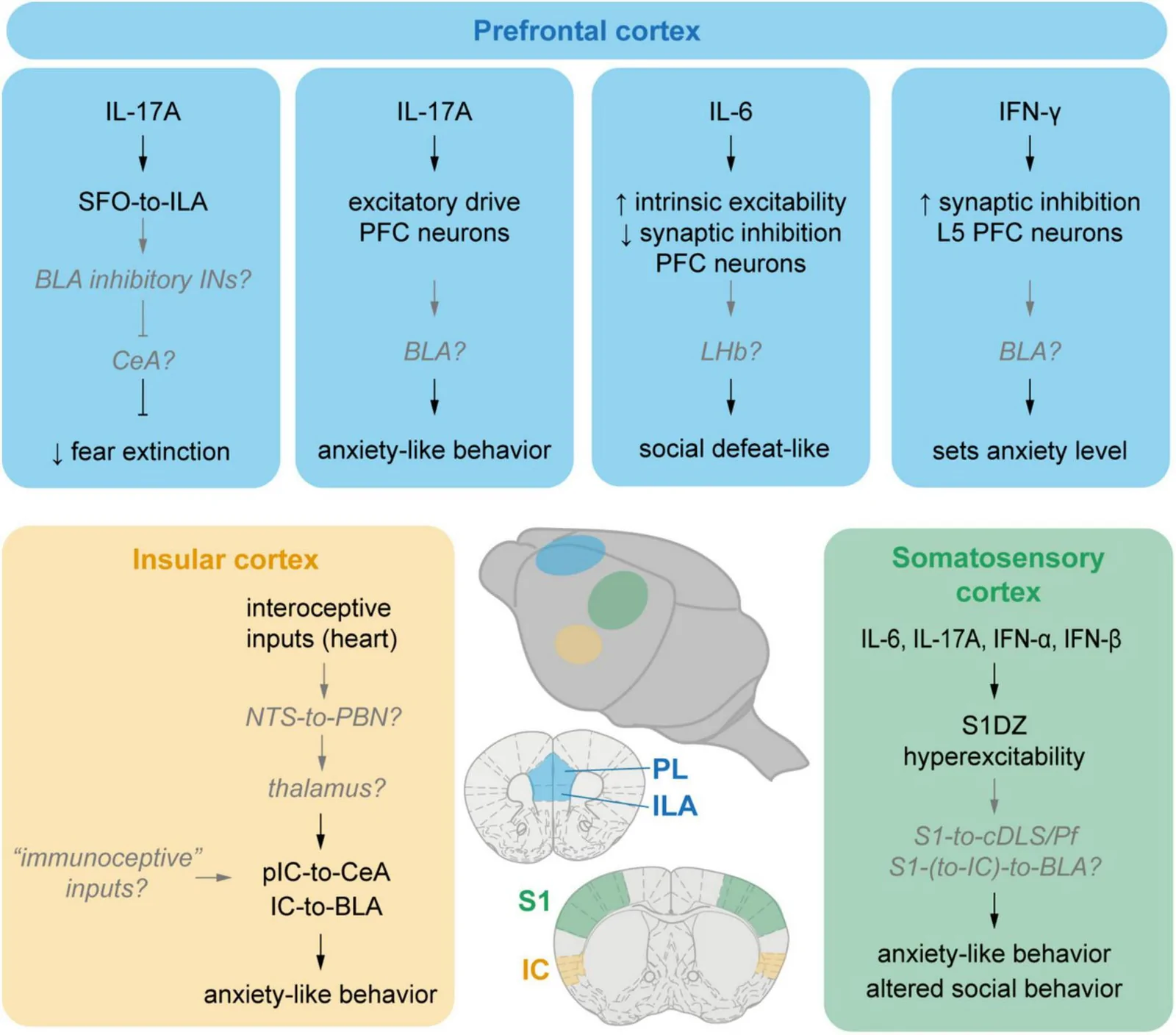
Cortical circuits through which peripheral inflammation influences affective behavior. Schematic summarizing how individual cytokines act on the prefrontal cortex (PFC), insular cortex (IC), and primary somatosensory cortex (S1) to alter affective behavior. Center: mouse brain shown in sagittal view (top) and coronal sections (bottom), indicating the prelimbic (PL) and infralimbic (ILA) subregions of the PFC, S1, and IC. Black arrows denote experimentally supported steps; gray arrows and gray italicized labels followed by a question mark denote proposed or not-yet-resolved connections. Prefrontal cortex (blue panels, left to right). (1) Interleukin-17A (IL-17A) acts in the subfornical organ (SFO), a circumventricular organ that projects to the infralimbic cortex (ILA), engaging downstream basolateral amygdala (BLA) inhibitory interneurons (INs) and the central nucleus of the amygdala (CeA) to modulate fear extinction. (2) IL-17A increases excitatory drive onto PFC neurons, which, potentially via PL-to-BLA projections, promotes anxiety-like behavior. (3) Interleukin-6 (IL-6) increases the intrinsic excitability and decreases the synaptic inhibition of PFC neurons (net disinhibition), which, potentially via projections to the lateral habenula (LHb), promotes “social defeat-like” behavior. (4) Interferon-gamma (IFN-γ) increases synaptic inhibition of layer 5 (L5) PFC pyramidal neurons, setting the level of anxiety-like behavior. Together, these panels illustrate that different cytokines perturb cortical excitatory/inhibitory balance in opposite directions yet converge on anxiety-like behavior. Insular cortex (orange panel). Interoceptive signals (e.g., from the heart), relayed through the nucleus of the solitary tract (NTS), together with putative “immunoceptive” (immune-related) inputs, are integrated in the IC; posterior insular cortex (pIC)-to-CeA and IC-to-BLA projections in turn drive anxiety-like behavior. Somatosensory cortex (green panel). Cytokines elevated by maternal immune activation, such as IL-6, IL-17A, interferon-alpha (IFN-α), and interferon-beta (IFN-β), produce hyperexcitability of the dysgranular zone of S1 (S1DZ), which, potentially via S1-to-BLA, S1-to-caudal dorsolateral striatum (cDLS), S1-to-the parafascicular nucleus of the thalamus and/or S1-to-IC-to-BLA projections, leads to anxiety-like and altered social behavior.

### 3.1 Prefrontal cortex

The prefrontal cortex (PFC) supports executive control and behavioral flexibility, integrating cognitive, emotional, and motivational information (Miller and Cohen, 2001; Rich and Shapiro, 2007). This role makes it a key interface at which peripheral inflammation can be translated into affective change. The PFC is well placed to receive immune signals, as its prelimbic (PL) and infralimbic (ILA) subregions lie close to the immunologically rich meninges (Frederick et al., 2022; Wu et al., 2025), and receive projections from CVOs. Both routes have been shown to deliver immune signals to the PFC (Alves de Lima et al., 2020; Allgire et al., 2026), but whether ascending interoceptive relays also do so remains untested.

This immune-to-PFC signaling has been shown to reshape PFC-dependent behavior. Allgire and colleagues showed that severe airway inflammation disrupts fear extinction (the suppression of learned fear in the absence of threat, whose failure defines anxiety and trauma-related disorders). This disruption occurs through IL-17A signaling in the subfornical organ (SFO), a CVO that projects to the ILA. Blocking IL-17A or silencing SFO-to-ILA projections restored normal extinction, confirming this circuit as a conduit between peripheral immunity and PFC function (Allgire et al., 2023, 2026).

PFC neurons also directly respond to cytokines, with effects that differ at homeostasis and under challenge. At homeostasis, meningeal γδ T cell-derived IL-17A engages IL-17Ra on PFC glutamatergic neurons, and neuronal deletion of the receptor reduces anxiety-like behavior (Alves de Lima et al., 2020). Interferon gamma (IFN-γ) increases inhibitory input onto pyramidal cells to set baseline anxiety levels (Garofalo et al., 2023). Under challenge, IL-6, elevated by systemic immune activation, leads to a social defeat phenotype. This occurs by altering the intrinsic excitability of PFC neurons and reducing inhibitory input onto pyramidal cells, promoting disinhibition (Garcia-Oscos et al., 2015; Esquivel-Rendón et al., 2019). Consistent with these findings, postmortem tissue from suicide victims, shows elevated proinflammatory cytokine and chemokine expression in the PFC (Pandey et al., 2012, 2021). Although correlational, these data indicate that the inflammatory signaling characterized in rodent PFC is also present in the human PFC of differing affective states. Together, these studies establish the PFC as a site where local inflammatory signaling sets affective tone at homeostasis and, under challenge, shifts it toward maladaptive states.

### 3.2 Insular cortex

The insular cortex (IC) is the primary cortical hub for interoceptive processing, integrating visceral and homeostatic signals with exteroceptive input and emotions (Gogolla, 2017). Although the exact pathways are not fully elucidated, “immune information” likely reaches the IC through the neural pathway relayed via the NTS, parabrachial nucleus, and thalamus (Gehrlach et al., 2020; Holt, 2022). Consistent with this, intestinal infection with *Campylobacter jejuni* induces c-Fos expression throughout the interoceptive network, including the vagal ganglia, NTS, and IC (Goehler et al., 2005).

In humans, the IC tracks inflammatory state across both experimental and clinical contexts. Human imaging shows increased activity and microstructural changes in the IC and other regions following experimental immune activation (Harrison et al., 2015; Kraynak et al., 2018). Notably, TNF-α neutralization therapy in patients with arthritis affects IC activity before it achieves anti-inflammatory effects in the joints, indicating that the IC may be among the earliest cortical regions to sense a change in immune state and/or inflammation-associated pain (Hess et al., 2011).

The IC is especially suited to link inflammation with affective dysregulation because interoceptive signals are actively incorporated into its evaluation of threat. During fear conditioning, cardiac feedback shapes IC activity to balance fear expression and extinction (Klein et al., 2021), and artificially imposed tachycardia activates the IC and heightens anxiety, an effect abolished by IC inhibition (Hsueh et al., 2023). The IC therefore treats bodily state as information about external threat, and raising heart rate alone is enough to shift that evaluation. Whether a change in immune state acts the same way as cardiac signals is untested. Such readouts are adaptive while the bodily signal is transient and informative. If inflammation is sustained, the same mechanism would report a threat that no longer exists and become maladaptive.

### 3.3 Primary somatosensory cortex

The primary somatosensory cortex (S1) represents sensory information from across the body (Penfield and Boldrey, 1937). Through its connections with the IC and amygdala, S1 integrates this information with affective and interoceptive signals, assigning emotional salience to sensory stimuli (particularly pain) and contributing to the generation and regulation of emotion (McDonald, 1998; Kropf et al., 2019). Altered sensitivity to environmental stimuli, heightened vigilance, and abnormal bodily sensations are common features of affective dysregulation, and structural and functional abnormalities in S1 are reported across psychiatric disorders (Qi et al., 2014; Cui et al., 2016; Schmaal et al., 2017; Minuzzi et al., 2018). S1 dysfunction may thus bias stimulus evaluation toward perceived threat, contributing to hypervigilance and maladaptive behavior. Border immunity acts on S1 during development: meningeal group 2 innate lymphoid cells release IL-13 onto inhibitory neurons to promote inhibitory synapse formation, shaping later social behavior (Barron et al., 2024). Whether the same interface conveys inflammatory signals to S1 in adulthood is unknown, and no ascending relay has been demonstrated for this region.

Although the route remains unclear, immune signaling can nonetheless alter cortical sensory processing itself, offering a mechanism by which immune activation influences affective behavior. The clearest example comes from maternal immune activation (MIA), in which prenatal immune challenge produces long-lasting cortical and behavioral abnormalities in offspring. MIA elevates multiple pro-inflammatory cytokines, including IL-17A, IL-6, and interferons, and yields offspring with increased anxiety-related behavior (Kim et al., 2024). Mechanistically, these abnormalities are linked to hyperexcitability in the dysgranular zone of S1 (S1DZ), a region involved in sensory integration (Shin Yim et al., 2017; Kalish et al., 2021). Activating excitatory pyramidal neurons in S1DZ is sufficient to reproduce the behavioral phenotype, whereas suppressing them rescues it. This is not limited to the MIA model, as deleting IL-17RA in S1DZ also reduces anxiety (Alves de Lima et al., 2020). Together, these studies causally link cytokine signaling in S1 to affective behavior. IL-17RA signaling sets anxiety levels at homeostasis, whereas developmental IL-17A exposure produces a lasting phenotype. Thus, the same pathway acts adaptively and maladaptively depending on when it is engaged.

## 4 Cortico-cortical and cortico-subcortical circuits

The behavioral consequences of inflammation-induced cortical changes depend on how these regions communicate with one another and with downstream targets. Direct evidence that inflammation alters cortico-cortical coupling is sparse and correlational. Altered S1-IC functional connectivity has been associated with elevated IL-6 in patients with bipolar disorder, suggesting that inflammation may interfere with the integration of sensory and interoceptive information (Minuzzi et al., 2018; Chen et al., 2020). The cortico-subcortical arm has been characterized almost entirely outside an inflammatory context. Both the PFC and IC are reciprocally connected with the amygdala, and dysfunction of these circuits is observed across anxiety disorders, PTSD, and specific phobia (Etkin and Wager, 2007). The pathways delineated below are therefore established routes by which cortical changes are translated into altered affective states, but mostly inferred conduits for inflammation.

### 4.1 PFC-amygdala

The PFC bidirectionally communicates with the amygdala (Ghashghaei et al., 2007). Both the PL and ILA project to the basolateral amygdala (BLA) but drive opposing outputs: PL facilitates fear expression, whereas ILA suppresses fear and supports extinction by reducing the responsiveness of central amygdala output neurons to BLA input (Quirk et al., 2003; Vidal-Gonzalez et al., 2006). Although this dichotomy simplifies a complex circuit, it offers a useful framework for interpreting how inflammation-related shifts in PFC activity translate into altered behavior.

No study has yet tested these circuits causally under peripheral inflammation. Allergic airway inflammation is associated with enhanced PFC-BLA connectivity (Dehdar et al., 2019), but causality has not been established. Airway inflammation also impairs fear extinction through the SFO-to-ILA pathway described above (Allgire et al., 2026), a behavior that depends on ILA projections to the BLA, but the amygdala output was not examined. Other sustained aversive states do engage the circuit: nerve injury elevates PL-to-BLA activity and drives comorbid anxiety-like behavior (Gao et al., 2023), and chronic stress disrupts PFC-amygdala functional connectivity in humans (Serrano et al., 2026). Whether inflammation acts on the same circuit, biasing PFC representations of internal states toward aversive coding (Hurley et al., 2023; Ma et al., 2024), remains to be tested.

### 4.2 IC-amygdala

The IC is also extensively and reciprocally connected with the amygdala, integrating the emotional significance of a stimulus and the state of the body so that internal states linked to past harm can predict future threat (Gehrlach et al., 2020). Projection-specific studies have made this relationship causal. Optogenetic manipulation of a posterior IC-to-central amygdala pathway bidirectionally alters anxiety-like behavior (Gehrlach et al., 2019), while anterior IC-to-BLA projections are activated during exploration of anxiogenic environments and by aversive stimuli, and are themselves anxiogenic (Nicolas et al., 2023). Together, these pathways may govern distinct aspects or temporal phases of the anxiety response. In the complete Freund’s adjuvant (CFA) inflammatory pain model, IC-BLA connectivity is reportedly enhanced (Wei et al., 2025). However, as for the PFC, the causality of the IC-BLA pathway for inflammation-induced anxiety has not been established. Because the IC-amygdala loop is driven by changes in bodily state (Hsueh et al., 2023), it is a plausible cortical route by which inflammation could bias interoceptive representations toward threat.

### 4.3 S1-subcortical circuits

S1 influences affective behavior through direct projections to the BLA and indirect cortico-cortical, including insular, cortico-striatal, and cortico-thalamic routes (McDonald, 1998; Chen et al., 2025; Adkins et al., 2026). Two of these have been tested under inflammation. S1-to-caudal dorsolateral striatum projections drive comorbid anxiety in the CFA model (Jin et al., 2020), and S1 projections to the parafascicular thalamus contribute to anxiety-like behavior after formalin-induced acute inflammatory pain (Ishikawa et al., 2023). The S1-to-BLA route remains untested under inflammation.

## 5 Discussion

We have traced a path from peripheral immune activation to the cortical circuits that govern affect. Once peripheral inflammation is initiated, the neural and humoral pathways give the brain a two-dimensional readout of peripheral immune activity. The neural arm preserves the anatomical origin of inflammation and relays it rapidly. The humoral arm lacks such spatial resolution but may report more faithfully the magnitude and duration of inflammation. These pathways engage brainstem nuclei, subcortical areas, and higher cortical regions, including the PFC, IC, and S1. Peripheral immune signals lead to altered excitability and connectivity that shift circuit outputs toward perceived threat.

Acutely, these processes are adaptive: hypervigilance and avoidance limit further exposure and prioritize recovery. When dysregulated, such as in chronic or recurrent inflammation, the same signaling turns maladaptive. In this framework, anxiety emerges from altered representations of internal physiological state: anxiety-prone individuals may over-attend to bodily sensations, biasing interoceptive predictions toward future threat and producing hypervigilance and avoidance (Paulus and Stein, 2006).

This traffic is not one-way. The IC, in particular, acts back on peripheral immunity. Reactivating ensembles tagged during zymosan-induced peritonitis and dextran sulfate sodium-induced colitis is sufficient to reinstate inflammation in the same peripheral tissues originally affected (Koren et al., 2021). The IC therefore both monitors peripheral immune state and contributes to its anticipatory regulation. Whether the circuits that bias affect toward threat overlap with those that regulate immunity is unresolved.

Almost all of the effects we discussed have been demonstrated after acute or developmental challenge. Whether they represent adaptive responses that resolve, or the early stages of maladaptive dysregulation, is largely untested. A central question for future work is which inflammatory conditions engage which circuits, and whether distinct immune challenges converge on shared substrates or recruit dedicated ones. Resolving this specificity is essential to explain why inflammation produces heterogeneous psychiatric presentations, and it may also be therapeutically useful. If discrete circuits mediate the affective consequences of inflammation, they offer targets for interrupting the path from immune activation to maladaptive affective dysregulation.
